# Supplementary material for: Three new species of Byrsopteryx Flint microcaddisflies from Peru (Insecta: Trichoptera) including DNA-based larval associations
Source: PeerJ. 2021 Dec 24;9:e12645. doi: 10.7717/peerj.12645 (PMC8711280; doi:10.7717/peerj.12645)
Supplement: Supplemental Information 2 [file peerj-09-12645-s002.pdf]

Supplementary Table 1. K2P distances among 20 COI sequences of nine *Byrsopteryx* species sampled.

	C. edwardsi HQ971758	*B. carioca* ENT0056 ♂	*B. espinhosa* ENT0005 ♂	*B. inti* ENT0702 ♂	*B. mamaocllo* ENT0703 ♂	*B. mamaocllo* ENT5519 larva	*B. mamaocllo* ENT5516 ♂	*B. esparta* ENT0123 ♂	*B. tapanti* HQ971757	*B. gomezi* AF436490	*B. gomezi* KX107513	*B. abrelata* ENT0068 ♂	*B. mancocapac* ENT0704 ♂	*B. mancocapac* ENT5408 larva	*B. mancocapac* ENT5410 larva	*B. mancocapac* ENT5494 ♂	*B. mancocapac* ENT5414 larva	*B. mancocapac* ENT5413 larva	*B. mancocapac* ENT5409 larva	*B. mancocapac* ENT5411 larva
C. edwardsi HQ971758		0.2264	0.2613	0.2661	0.2495	0.2641	0.2623	0.2319	0.2306	0.2504	0.2258	0.2528	0.2175	0.2256	0.2199	0.2331	0.2268	0.2298	0.2287	0.2456
B. carioca ENT0056 ♂	0.2264		0.2356	0.2548	0.2404	0.2469	0.2483	0.2216	0.2565	0.2388	0.2388	0.2161	0.2435	0.2387	0.2335	0.2376	0.2338	0.2166	0.2400	0.2370
B. espinhosa ENT0005 ♂	0.2613	0.2356		0.1846	0.2060	0.2158	0.2169	0.2084	0.2156	0.2082	0.2017	0.1995	0.2077	0.1952	0.2151	0.2014	0.2024	0.1960	0.2092	0.2102
B. inti ENT0702 ♂	0.2661	0.2548	0.1846		0.1675	0.1492	0.1499	0.1868	0.1752	0.1691	0.1810	0.2127	0.2259	0.2272	0.2310	0.2284	0.2181	0.2066	0.2202	0.2294
B. mamaocllo ENT0703 ♂	0.2495	0.2404	0.2060	0.1675		0.0074	0.0049	0.1949	0.2208	0.2199	0.2211	0.2363	0.2211	0.2205	0.2319	0.2320	0.2216	0.2186	0.2259	0.2215
B. mamaocllo ENT5519 larva	0.2641	0.2469	0.2158	0.1492	0.0074		0.0000	0.2068	0.2168	0.2204	0.2231	0.2606	0.2198	0.2205	0.2229	0.2172	0.2122	0.2064	0.2151	0.2215
B. mamaocllo ENT5516 ♂	0.2623	0.2483	0.2169	0.1499	0.0049	0.0000		0.2079	0.2180	0.2210	0.2210	0.2589	0.2210	0.2205	0.2241	0.2184	0.2135	0.2079	0.2163	0.2215
B. esparta ENT0123 ♂	0.2319	0.2216	0.2084	0.1868	0.1949	0.2068	0.2079		0.1684	0.1562	0.1585	0.2291	0.2390	0.2546	0.2514	0.2489	0.2409	0.2322	0.2427	0.2514
B. tapanti HQ971757	0.2306	0.2565	0.2156	0.1752	0.2208	0.2168	0.2180	0.1684		0.1228	0.1336	0.2127	0.2178	0.2181	0.2279	0.2223	0.2036	0.2073	0.2080	0.2112
B. gomezi AF436490	0.2504	0.2388	0.2082	0.1691	0.2199	0.2204	0.2210	0.1562	0.1228		0.0000	0.2202	0.2340	0.2175	0.2370	0.2346	0.2307	0.2120	0.2265	0.2105
B. gomezi KX107513	0.2258	0.2388	0.2017	0.1810	0.2211	0.2231	0.2210	0.1585	0.1336	0.0000		0.2249	0.2178	0.2175	0.2199	0.2196	0.2108	0.2043	0.2129	0.2105
B. abrelata ENT0068 ♂	0.2528	0.2161	0.1995	0.2127	0.2363	0.2606	0.2589	0.2291	0.2127	0.2202	0.2249		0.2071	0.2049	0.2038	0.1985	0.2023	0.1908	0.2017	0.1915
B. mancocapac ENT0704 ♂	0.2175	0.2435	0.2077	0.2259	0.2211	0.2198	0.2210	0.2390	0.2178	0.2340	0.2178	0.2071		0.0000	0.0112	0.0096	0.0102	0.0074	0.0067	0.0061
B. mancocapac ENT5408 larva	0.2256	0.2387	0.1952	0.2272	0.2205	0.2205	0.2205	0.2546	0.2181	0.2175	0.2175	0.2049	0.0000		0.0137	0.0055	0.0082	0.0066	0.0054	0.0061
B. mancocapac ENT5410 larva	0.2199	0.2335	0.2151	0.2310	0.2319	0.2229	0.2241	0.2514	0.2279	0.2370	0.2199	0.2038	0.0112	0.0137		0.0134	0.0099	0.0047	0.0059	0.0092
B. mancocapac ENT5494 ♂	0.2331	0.2376	0.2014	0.2284	0.2320	0.2172	0.2184	0.2489	0.2223	0.2346	0.2196	0.1985	0.0096	0.0055	0.0134		0.0061	0.0073	0.0061	0.0000
B. mancocapac ENT5414 larva	0.2268	0.2338	0.2024	0.2181	0.2216	0.2122	0.2135	0.2409	0.2036	0.2307	0.2108	0.2023	0.0102	0.0082	0.0099	0.0061		0.0019	0.0034	0.0030
B. mancocapac ENT5413 larva	0.2298	0.2166	0.1960	0.2066	0.2186	0.2064	0.2079	0.2322	0.2073	0.2120	0.2043	0.1908	0.0074	0.0066	0.0047	0.0073	0.0019		0.0000	0.0000
B. mancocapac ENT5409 larva	0.2287	0.2400	0.2092	0.2202	0.2259	0.2151	0.2163	0.2427	0.2080	0.2265	0.2129	0.2017	0.0067	0.0054	0.0059	0.0061	0.0034	0.0000		0.0000
B. mancocapac ENT5411 larva	0.2456	0.2370	0.2102	0.2294	0.2215	0.2215	0.2215	0.2514	0.2112	0.2105	0.2105	0.1915	0.0061	0.0061	0.0092	0.0000	0.0030	0.0000	0.0000	
